# Thymol and Carvacrol Against *Echinococcus* spp.: Experimental Evidence, Mechanistic Insights, and Translational Perspectives

**DOI:** 10.1007/s11686-026-01302-4

**Published:** 2026-05-11

**Authors:** Elodie Mimi Megnigueu, Sami Simsek, Figen Celik, Siméon Fogué Kouam, Dieudonné Ndjonka

**Affiliations:** 1 Department of Biological Sciences, Faculty of Sciences, University of Ngaoundere, P.O. Box 454, Ngaoundéré, Cameroon; 2 Department of Parasitology, Faculty of Veterinary Medicine, University of Firat, 23119 Elazig, Türkiye; 3 Department of Chemistry, Higher Teacher Training College, University of Yaoundé I, P.O. Box 47, Yaoundé, Cameroon

**Keywords:** *Echinococcus granulosus*, *Echinococcus multilocularis*, Thymol, Carvacrol

## Abstract

**Purpose:**

Echinococcosis remains a major neglected zoonosis with limited therapeutic options. Thymol and carvacrol have emerged as experimental anti-echinococcal candidates, yet their mechanistic basis, translational relevance, and major limitations including the absence of clinical evidence remain incompletely defined. This review critically evaluates available evidence on their anti-echinococcal activity, examines proposed mechanisms of action, and identifies key barriers that must be addressed for therapeutic development.

**Methods:**

A substantial body of experimental research has been conducted to evaluate their efficacy, and this narrative review synthesizes and critically examines available evidence regarding their anti-echinococcal activity and proposed mechanisms of action.

**Results:**

Available experimental evidence indicates that both isomers exhibit anti-echinococcal activity in in vitro and in vivo models involving *Echinococcus granulosus sensu lato* and *Echinococcus multilocularis*. Their effects are mainly associated with tegumental membrane disruption, accompanied by ultrastructural alterations, while evidence supporting apoptosis-like pathways remains preliminary and incompletely validated. Experimental studies indicate synergistic or additive interactions with albendazole, suggesting possible mechanistic complementarity, although the basis of these interactions remains incompletely defined.

**Conclusion:**

Although experimental evidence supports membrane-disruptive and anti-echinococcal activity of thymol and carvacrol, current evidence remains largely preclinical and insufficient for therapeutic translation, particularly in the absence of clinical research data. Major uncertainties including poor aqueous solubility, limited bioavailability, lack of intracystic pharmacokinetic data, methodological heterogeneity, and incompletely validated mechanistic pathways remain unresolved. These monoterpenoid phenols should therefore be regarded as promising experimental candidates rather than clinically translatable agents, pending rigorous preclinical validation.

**Graphical Abstract:**

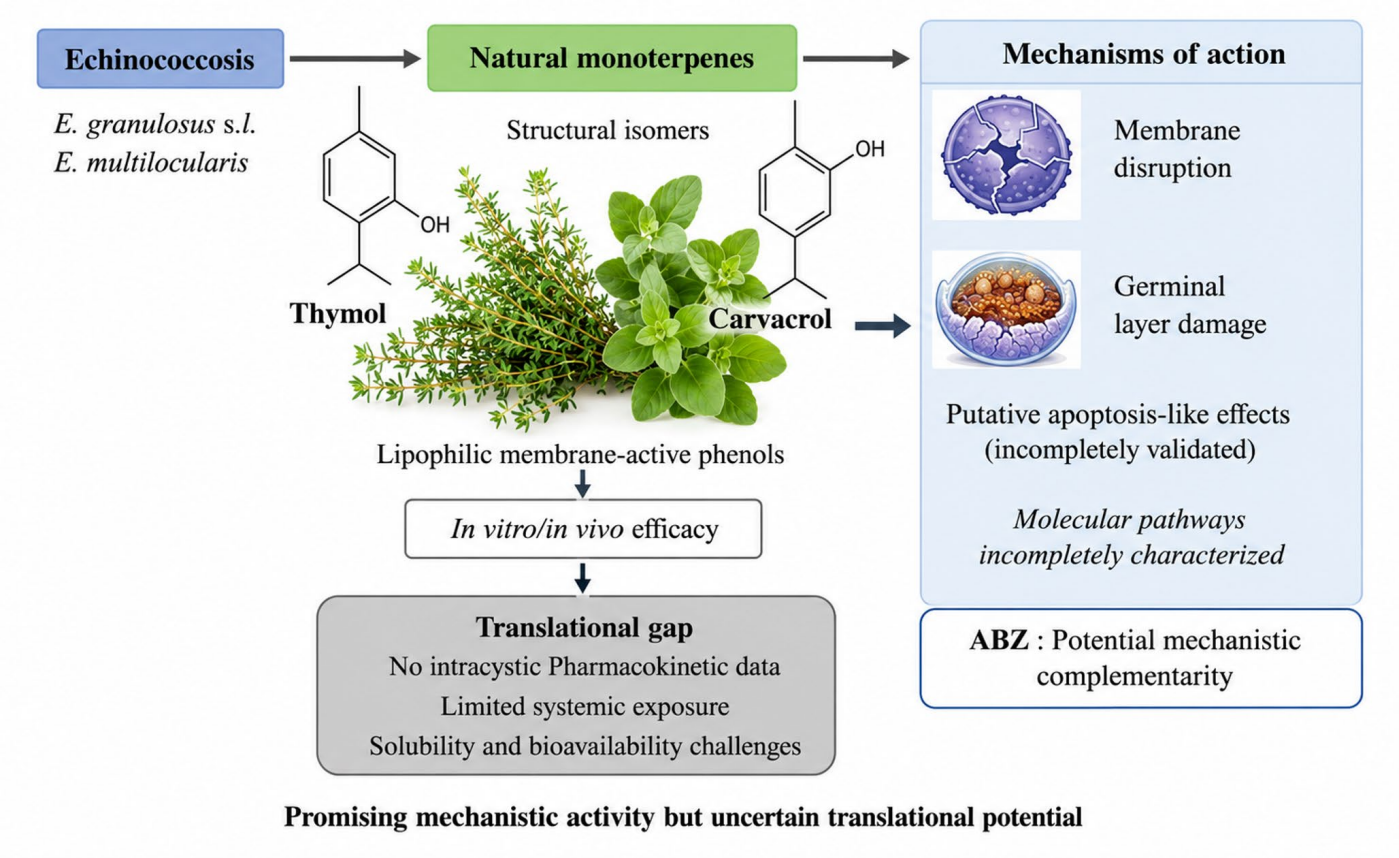

## Introduction

Echinococcosis is a neglected zoonotic helminthic disease caused by the larval stages of cestodes of the genus Echinococcus. The *Echinococcus granulosus sensu lato* (s.l.) complex is primarily associated with cystic echinococcosis (CE), whereas *Echinococcus multilocularis*, a distinct species, causes alveolar echinococcosis (AE), a more infiltrative and clinically severe form of the disease.

Human infection occurs through accidental ingestion of embryonated eggs shed by infected definitive hosts [1]. CE has a global distribution, whereas AE is largely restricted to the Northern Hemisphere and causes more severe disease [2]. Transmission dynamics and epidemiological risk vary among *Echinococcus* species [3]. Beyond its broad distribution, echinococcosis imposes a substantial global health and socioeconomic burden. CE affects hundreds of thousands of people worldwide and contributes considerably to disability-adjusted life years (DALYs) lost annually, while AE, although less common, is associated with high morbidity and potentially fatal outcomes if untreated. Chronic hepatic and pulmonary involvement, invasive growth in AE, long treatment courses, and frequent recurrence contribute to major clinical burden in affected populations. In addition, losses related to livestock production and control programs further amplify the impact of the disease in endemic regions. This burden underscores the continued need for improved therapeutic strategies [3].

In the *E. granulosus s.l.* transmission cycle, domestic dogs act as principal definitive hosts in livestock-associated cycles, whereas *E. multilocularis* mainly circulates in sylvatic cycles involving wild canids and rodents, with occasional domestic transmission contributing to human exposure [4, 5]. CE and AE differ markedly in pathology and clinical behavior. CE typically forms well-defined cystic lesions, mainly in the liver and lungs, whereas AE shows infiltrative, tumor-like growth primarily in the liver and is associated with more severe disease [6]. Given the substantial burden of echinococcosis and limitations of current interventions, improved therapies remain a priority. Treatment relies on surgery when feasible or chemotherapy with benzimidazoles such as albendazole and mebendazole [6, 7]. However, benzimidazoles are often only parasitostatic in AE, require prolonged administration, and may be associated with poor compliance, adverse effects, limited cyst penetration, and treatment failure in a proportion of cases [8–11]. These limitations support exploration of alternative pharmacological strategies, including bioactive compounds derived from medicinal plants.

Medicinal plants are an important source of bioactive compounds. Among these, essential oils rich in terpenoid constituents have attracted interest as potential alternative or adjunct anti-echinococcal agents, supported by experimental in vitro and in vivo evidence [12–16].

Among essential oil constituents, thymol and carvacrol have attracted attention as experimental anti-echinococcal candidates because of broad antiparasitic activity, membrane-directed multi-target effects, potential complementarity with benzimidazoles, and favorable safety characteristics, including GRAS status. Unlike slow-acting parasitostatic benzimidazoles, these monoterpenoid phenols may offer distinct pharmacodynamic advantages in experimental settings and may have value not only through direct efficacy, but also through mechanism-based complementarity with existing therapies [17, 18, 19, 44]. However, evidence in echinococcosis remains entirely preclinical, with no clinical trials or human efficacy data currently available.

In this context, this narrative review critically synthesizes current evidence on the anti-echinococcal activity of thymol and carvacrol, examines proposed mechanisms, and identifies translational knowledge gaps relevant to therapeutic development.

## Materials and Methods

### Literature Search Strategy

Although the present work was designed as a narrative review, a structured and reproducible literature search was conducted to enhance transparency and reduce potential selection bias. Relevant publications were retrieved from PubMed, ScienceDirect, and Google Scholar, with the last search conducted on 31 December 2025.

The search strategy combined compound-related, parasite-related, and experimental-context terms using Boolean operators. The core search string applied in PubMed was: (“thymol” OR “carvacrol”) AND (“*Echinococcus granulosus*” OR “*Echinococcus multilocularis*” OR “echinococcosis” OR “hydatid cyst”) AND (“protoscoleces” OR “metacestodes” OR “in vitro” OR “in vivo” OR “albendazole” OR “combination therapy”). Equivalent keyword combinations were adapted for ScienceDirect and Google Scholar. Given the broader indexing scope of these platforms, the screening process was limited to articles ranked by relevance. Only original experimental studies directly evaluating thymol and/or carvacrol against *Echinococcus* spp. were considered eligible.

The PubMed search yielded 22 records. After title and abstract screening, 13 studies met the predefined inclusion criteria and were included in the qualitative synthesis, while nine records were excluded. Searches in ScienceDirect and Google Scholar, as well as manual screening of reference lists of included articles, did not identify any additional eligible studies.

Eligible studies were (i) original experimental investigations (in vitro and/or in vivo), (ii) evaluating isolated thymol and/or carvacrol, alone or with ABZ, (iii) assessing activity against *E. granulosus* or *E. multilocularis*, (iv) reporting quantitative or semi-quantitative efficacy outcomes. Studies were excluded if they (i) were review articles or conference abstracts, (ii) focused on unrelated parasitic species, (iii) investigated complex essential oils without clearly attributing observed effects to thymol or carvacrol. Screening and data extraction were performed by one reviewer, this represents a potential source of selection bias and should be considered a limitation of the present narrative review. Given the heterogeneity in experimental designs, concentration units, and outcome measures, findings were synthesized qualitatively rather than through meta-analysis. Because this work was structured as a narrative review, no formal risk-of-bias scoring framework (e.g., SYRCLE, ROBINS-I) was applied at the individual study level. This should be considered a limitation when interpreting the strength of the evidence. Nevertheless, methodological heterogeneity, model-specific limitations, and variability in outcome measures were critically considered in the qualitative synthesis. In addition, the focused database selection and narrative review design should be considered when interpreting the comprehensiveness of the evidence base.

### Chemical and Biological Properties of Thymol and Carvacrol

**Fig. 1:**
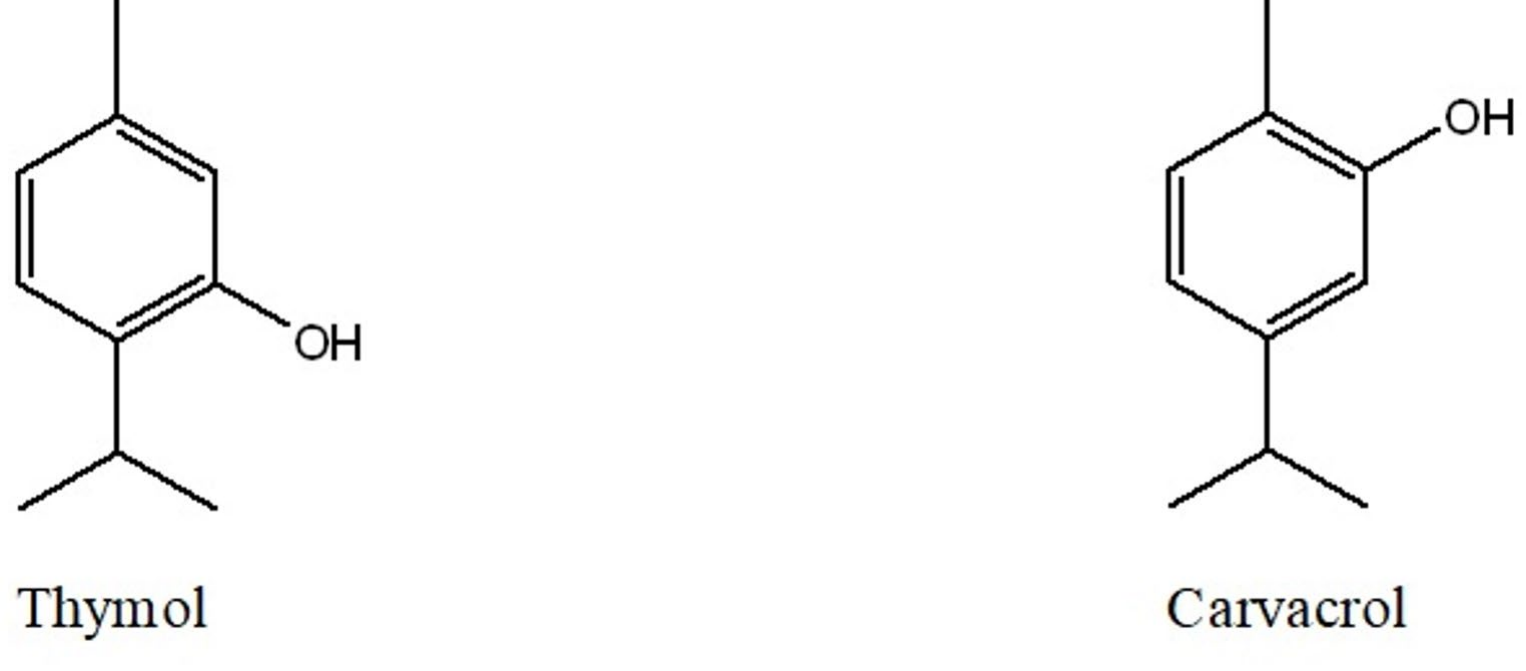
Chemical structures of thymol and carvacrol illustrating positional isomerism of the hydroxyl group on the aromatic ring

Thymol (5-methyl-2-isopropylphenol) and carvacrol (2-methyl-5-isopropylphenol) are structural isomers belonging to the phenolic monoterpenoid class of essential oil constituents. Although they share the same molecular formula (C_10_H_14_O), they differ in the position of the hydroxyl group on the aromatic ring (Fig. 1), a subtle variation that influences their physicochemical and biological behavior [19, 20].

These compounds are major bioactive constituents of essential oils derived from aromatic plants of the Lamiaceae family, such as *Thymus vulgaris* (thyme), *Origanum vulgare* (oregano), *Zataria multiflora*, and *Satureja hortensis* (savory). Thymol predominates in *Thymus* species, whereas carvacrol is frequently found in *Origanum* and *Satureja* species [21, 22]. Their relative concentrations vary according to plant genotype, ecological conditions, and extraction methods [23].

Thymol and carvacrol share physicochemical properties such as lipophilicity and volatility that favor membrane partitioning and permeabilization [24]. Their phenolic structure underlies these interactions, while the slightly greater hydrophobicity of carvacrol may contribute to its higher biological potency [25, 26].

Beyond their chemical similarities, thymol and carvacrol exhibit diverse biological activities linked to their phenolic structure, including membrane disruption, redox modulation, and enzyme inhibition [27]. They also display antioxidant and broad antimicrobial activities relevant to their pharmacological potential [28, 29]. Both compounds exhibit broad antibacterial activity through membrane destabilization and metabolic interference [30].

These effects are often mediated by membrane permeabilization, leakage of intracellular constituents, and interference with essential metabolic pathways [19, 31]. Moreover, both compounds inhibit biofilm formation and disrupt established biofilms, which enhances their antimicrobial potency and broadens their therapeutic relevance, particularly in the context of persistent infections [32, 33].

Both compounds exhibit antifungal activity against *Candida spp* and filamentous fungi such as *Aspergillus niger*, *Trichoderma viride*, *Aspergillus flavus*, and *Penicillium rubrum* [34, 29]. Antiviral effects have been observed mainly in enveloped viruses, where these monoterpenes compromise viral envelope integrity and interfere with replication processes [35, 36].

These compounds also modulate inflammatory responses through the downregulation of pro-inflammatory mediators, such as nitric oxide, prostaglandins, and cytokines like TNF-α, IL-1β, and IL-6 [37, 38]. Mechanistically, they inhibit activation of key signaling pathways such as NF-κB and MAPK, thereby suppressing the transcription of genes involved in inflammation and oxidative stress. Their combined antioxidant and anti-inflammatory properties support therapeutic potential in managing chronic inflammatory and infectious diseases [17].

Building on these multifaceted activities, thymol and carvacrol have shown antiparasitic activity against diverse helminth and protozoan species. Their efficacy has been attributed to mechanisms similar to those observed in antimicrobial contexts, including the disruption of parasite membrane integrity, induction of oxidative stress, and interference with essential enzymatic and metabolic pathways. Several studies indicate inhibitory effects of thymol and carvacrol on viability and motility in *Trypanosoma*, *Plasmodium*,* Giardia*,* Leishmania*,* Cryptosporidium*, and *Echinococcus* species, accompanied by structural damage to parasite membranes and intracellular organelles [39, 40, 41, 42, 43].

In experimental echinococcosis models, both compounds show dose- and time-dependent protoscolicidal activity linked to membrane-targeting effects. Their lipophilicity may support cyst penetration, while ancillary antioxidant and anti-inflammatory properties may contribute to host tissue protection.

### Toxicity and Safety Profile of Thymol and Carvacrol

Thymol and carvacrol, traditionally used as flavoring agents in food products, are classified as *Generally Recognized as Safe* (GRAS) by the United States Food and Drug Administration (FDA) [44]. At low concentrations, these compounds are considered safe and exhibit multiple biological activities. Their ability to interact with multiple cellular targets through distinct mechanisms of action has contributed to their extensive biomedical applications [45, 46].

**Fig. 2:**
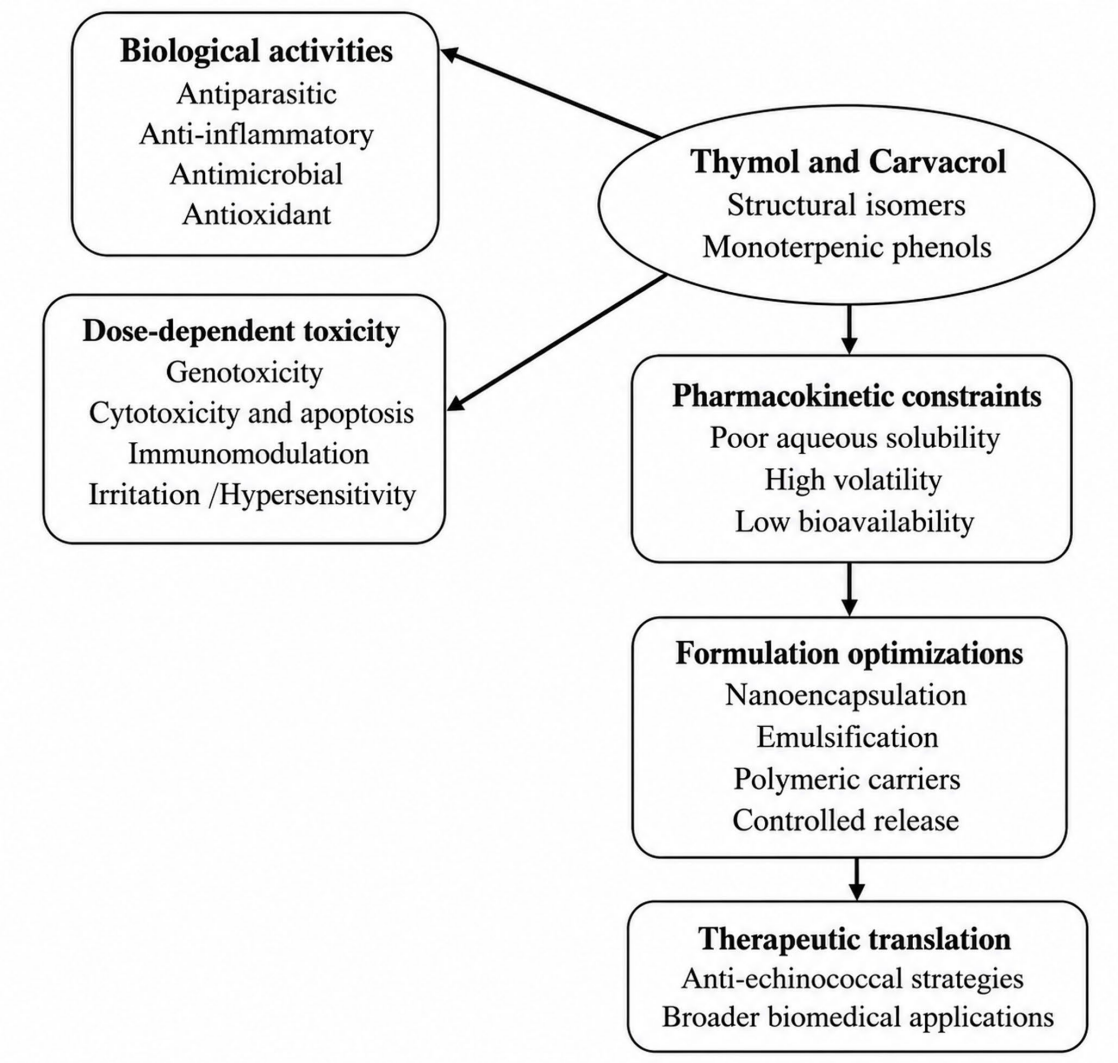
Translational framework of thymol and carvacrol: biological activities, toxicological considerations, and formulation strategies. This schematic illustrates the progression from established biological activities of thymol and carvacrol to their potential therapeutic application in echinococcosis

Despite their broad therapeutic potential, these compounds present several toxicological and pharmacokinetic limitations (Fig. 2). At high concentrations, both compounds have been associated with mutagenic and genotoxic effects. Carvacrol induces genotoxic effects in intestinal epithelial (Caco-2) cells, including DNA structural alterations [47]. Moreover, carvacrol alone or in combination with thymol has been show to induce cytotoxicity and apoptosis in Caco-2 cells, indicating potential risks for intestinal health [48]. Both compounds may also cause skin and eye irritation, and in some cases, hypersensitivity reactions such as allergic contact dermatitis [49, 50].

Available acute and subacute toxicity studies suggest that thymol and carvacrol are relatively well tolerated in experimental models, although toxicity remains dose- and route-dependent. Available acute toxicity data indicate route- and species-dependent LD50 values for carvacrol, including approximately 810 mg/kg orally in rats, 680 mg/kg subcutaneously in mice, 73–80 mg/kg intraperitoneally or intravenously in dogs, 310 mg/kg intravenously in dogs, and 2700 mg/kg by dermal exposure in rabbits [51]. These toxicity data indicate substantial species- and route-dependent variability, suggesting caution in cross-species extrapolation. Reliable estimation of a therapeutic index is currently not feasible because directly comparable efficacy dose metrics and standardized pharmacodynamic endpoints are lacking across available studies. For the same reason, extrapolation of potential human LD50 ranges would be speculative and is not currently evidence-based.

Thymol exhibits dose-dependent toxicity, including acute lethal effects in mice, whereas its acetate derivative shows reduced toxicity, suggesting that structural modification may improve its safety profile [52]. Thymol can also modulate immune responses and affect immune cell viability, raising concerns about potential immunotoxicity [53]. High doses have been linked to developmental abnormalities in chicken embryos [54], while in vitro studies revealed weak estrogenic and mutagenic activity below genotoxicity thresholds. Overall, thymol appears to induce tissue- specific and developmental toxicity at high doses, yet exhibits a relatively low genotoxic risk under controlled exposure conditions.

Additionally, the hydrophobic nature of thymol and carvacrol limits their aqueous solubility and bioavailability, potentially restricting systemic applications. To overcome these limitations, formulation strategies including nanoencapsulation, emulsification, and polymeric delivery systems have been developed to reduce volatility, enhance solubility, improve physicochemical stability, and enable controlled release [49, 55]. Such approaches may mitigate cytotoxicity while preserving biological activity.

Overall, although thymol and carvacrol have favorable safety profiles at controlled doses, careful consideration of dosage, administration route, and formulation is essential for their potential therapeutic applications, including in anti-echinococcal strategies. Notably, the differential toxicity profiles of these structural isomers underscore the importance of structural determinants in modulating biological and toxicological responses. Further integration of mechanistic toxicology and pharmacokinetic modeling will be critical to more precisely define their therapeutic window and optimize their translational development. Cross-species variability and lack of directly comparable efficacy dose metrics currently preclude reliable estimation of a therapeutic index.

### Anti-echinococcal Activities of Thymol and Carvacrol

Before examining the available experimental evidence, it is important to consider how in vitro and in vivo approaches contribute to our understanding of the anti-echinococcal potential of thymol and carvacrol. Given the chronic nature of echinococcosis and the limitations associated with current BMZ based therapies, the identification of novel or adjunctive compounds necessitates a stepwise validation strategy. In vitro assays remain the primary and most accessible investigative tools, offering rapid and reproducible insights into scolicidal and cysticidal activities under controlled conditions. In contrast, *in vivo assays*, typically carried out using animal models experimentally infected with protoscoleces or metacestodes, provide a more biologically comprehensive and clinically relevant context. This reflects the complex interactions between host and parasite, as well as the pharmacodynamics. Together, these complementary approaches build a rigorous and translational foundation for evaluating therapeutic relevance against *E. granulosus s.l.* and *E. multilocularis*. Interpretation of efficacy across studies requires caution due to heterogeneity in parasite stages examined, viability readouts (eosin exclusion, methylene blue, motility, morphological scoring), exposure durations, and concentration metrics. These differences limit direct cross-study comparisons and preclude robust quantitative conclusions regarding relative efficacy.

### In vitro Activity of Thymol and Carvacrol

Thymol is the most extensively investigated of these two phenolic monoterpenes in the context of echinococcosis. Multiple studies consistently demonstrate a potent protoscolicidal and cysticidal effect in clear, dose- and time-dependent manners. Studies [40, 56, 57, 58] have documented progressive morphological degeneration of protoscoleces following thymol exposure, including soma contraction, blebbing, microtriche shedding, vacuolization, and collapse of the germinal layer. Similar destructive alterations have been observed in *E. multilocularis* metacestodes, in which thymol disrupts membrane integrity and impairs vesicular architecture.

Carvacrol demonstrates a comparable antiparasitic profile. In vitro assays reveal pronounced ultrastructural damage in protoscoleces, such as tegumental disruption, microtriches shedding, vacuolization, and metabolic collapse [59, 60]. In *E. multilocularis*, carvacrol induces structural alterations in metacestode vesicles, although apparent efficacy may vary with parasite developmental stage and experimental conditions [61]. These findings indicate that both isomeric monoterpenes exert rapid and extensive structural damage across distinct parasite stages. Notably, thymol and carvacrol also demonstrate synergistic interactions with ABZ. Combination assays reveal enhanced parasite degeneration, accelerated germinal layer collapse, and greater reductions in viability compared with monotherapy [62, 63]. Such synergism is particularly relevant given the limitations associated with benzimidazole-based treatments, suggesting potential adjunctive therapeutic strategies.

In addition, a growing body of evidence underscores significant synergistic or additive interactions when thymol and carvacrol are administered together. A previous study [64] found that mixtures of both compounds produced more rapid and extensive tegumental disruption and structural collapse in protoscoleces than either monoterpene alone. More recent investigations demonstrate that different ratios of thymol and carvacrol inflict severe ultrastructural damage in metacestodes, including microtriches loss, vesiculation, and reduced cyst turgidity, culminating in complete parasite death [65]. Table 1 summarizes in vitro assays evaluating thymol and carvacrol, while also illustrating heterogeneity in experimental designs, viability endpoints, and reported efficacy across parasite stages.

**Table 1:** in vitro anti-echinococcal activity of thymol and carvacrol

Compound	Species (Stages)	Conc. (Original)	Approx. Conc (µM)	Exposure time	Assay/ Endpoint	Main outcomes	Comparison with reference drug	References
Thymol	*E. granulosus* (Protoscoleces)	100–250 µg/mL	670–1675	2–20 min	Viability: Methylene blue. Ultrastructure: SEM/TEM	Protoscoleces viability: Reduced to 1.3% at 250 µg/mL within 2 min; rapid tegumental damage	Not evaluated	Elissondo et al. [56]
Thymol + Carvacrol	*E. granulosus* (Protoscoleces)	12.5–100 µg/mL	83.8–670	5–30 min	Viability: Eosin exclusion test	Complete loss of protoscoleces viability at 100 µg/mL within 5 min;	More effective than 20% hypertonic saline and silver nitrate	Mahmoudvand et al. [64]
Thymol	*E. granulosus* (Protoscoleces)	1–50 µg/mL	6.7–333	5 days	Viability: Eosin exclusion	Complete loss of viability at 50 µg/mL	More rapid and potent than ABZ (800 µg/mL)	Yones et al. [59]
Carvacrol	*E. multilocularis* (Metacestodes)	12.5–850 µM	Not applicable	5 days	Viability: Alamar blue assay	Limited activity at ≤ 100 µM; vesicle damage and reduced viability at ≥ 150 µM; minor structural changes	Not evaluated	Hizem et al. [61]
Thymol	*E. multilocularis* (Metacestodes)	12.5–100 µM	Not applicable	5 days	Viability: Alamar blue assay	Weak activity at tested concentrations	Not evaluated	
Thymol	*E. granulosus* (Germinal layer cells)	1–10 µg/mL	6.7–67	7 days	Viability: Trypan blue and morphology. Ultrastructure: SEM.	63% reduction in cell viability at 5 µg/mL; marked cell collapse	Not evaluated	Albani et al. [58]
Carvacrol + ABZ	*E. multilocularis* *(*Metacestodes)	CA: 10 µg/mL; ABZ: 10 µg/mL 1–10 µg/mL Combination	67 67 6.7–67	10 days	Viability: Morphology. Ultrastructure: SEM	Moderate activity of CA alone; combination (10 µg/mL CA + 1 µg/mL ABZ) reduced viability to 23% at day 10; germinal layer collapse	CA less than ABZ. Combination significantly more effective than ABZ alone	Lopez et al. [63]
Thymol	*E. multilocularis* (Protoscoleces)	10 µg/mL	67	10–18 days	Viability: Methylene blue and morphology. Ultrastructure: SEM	Viability reduced to 33% with ABZ + thymol (10 µg/mL each) at day 18; early tegument alterations (day 3); severe ultrastructural damage	Combination more effective than either treatment alone	Albani and Elissondo [62]
	*E. multilocularis* (Metacestodes)	10 µg/mL	67	10–18 days	Viability:GGT activity. Ultrastructure : SEM	Complete vesicle disruption and germinal layer distortion with combination; increased GGT activity at day 3; SEM confirmed severe structural damage.	Combination induced greater damage and higher GGT release than monotherapies	
Carvacrol	*E. granulosus* (Protoscoleces)	1–10 µg/mL	6.7–67	60 days	Viability: Methylene blue; Apoptosis: TUNEL; Ultrastructure: SEM/ TEM	viability reduced to 17% at day 6 and 0% at day 60 (10 µg/mL); severe tegumental damage; DNA fragmentation detected after 24 h	Not evaluated	Fabbri et al. [60]
Carvacrol + Thymol	*E. granulosus* *(*Protoscoleces)	Ratios 9:1; 5:5; 1:9	Not applicable; mixture ratios	Up to 60 days	Viability: Methylene blue. Ultrastructure: SEM	Viability decreased to 40 − 38% at day 6 and 0% at day 60 (9:1 and 5:5); severe tegumental damage	Not evaluated	Albani et al. [65]
Thymol	*E. granulosus* *(*Protoscoleces)	10 µg/mL	67	Up to 72 days	Viability: Methylene blue; GGT activity; A poptosis: TUNEL	Viability reduced to 55% at day 12 and 0% at day 72; increased GGT activity; ultrastructural damage; DNA fragmentation detected	Not evaluated	Pensel et al. [57]
Thymol	*E. granulosus* (Protoscoleces)	10 µg/mL	67	80 days	Viability: Methylene blue; motility; Ultrastructure: SEM/TEM	Progressive loss of motility; viability reduced to 53.5% (day 12) and 0% (day 80); tegumental damage	Not evaluated	Elissondo et al. [40]

Approximate µM values were calculated based on the molecular weight of thymol and carvacrol (150.22 g/mol); SEM: scanning electron microscopy; TEM: transmission electron microscopy; TUNEL: terminal deoxynucleotidyl transferase dUTP nick end labeling; GGT: gamma-glutamyl transferase; ABZ: albendazole; CA: carvacrol; Conc: concentration. Due to differences in parasite stage, endpoint definitions and exposure protocols, efficacy comparisons across studies should be interpreted cautiously

In vitro studies consistently highlight the potent scolicidal and cysticidal activities of thymol and carvacrol, mediated through tegumental disruption, membrane destabilization, and metabolic collapse. Their broad ranging activity across developmental stages, coupled with documented synergism with ABZ, positions thymol and carvacrol as promising natural candidates for adjunctive therapies in the management of echinococcosis.

### In vivo Activity of Thymol and Carvacrol

Several experimental studies using murine infection models have assessed thymol and carvacrol, administered either as monotherapies or in combination with ABZ, as summarized in Table 2. Thymol has been shown to markedly reduce the infectivity of *E. granulosus* protoscoleces. In a study [57], thymol pretreated protoscoleces failed to establish infection in mice. Another study [66] reported that repeated oral dosing of thymol reduced cyst weight and induced extensive ultrastructural alterations in the germinal layer of *E. granulosus*-infected mice, without evidence of systemic toxicity. In *E. multilocularis* models, thymol produced notable reductions in cyst development, and its combination with ABZ yielded greater inhibition of parasite growth and more pronounced morphological damage than either treatment alone [67]. These findings underscore the intrinsic efficacy of thymol and its potential synergistic interaction with ABZ.

**Table 2:** in vivo studies of thymol and carvacrol in experimental echinococcosis

Parasite	Animal model	Treatment	Dose	Duration	Assay/endpoint	Main outcomes	Compared with ABZ	Reference
*E. granulosus*	CF-1 Mice	Thymol	40 mg/kg	Once daily for 20 days or twice daily for 10 days	Cyst weight measurement; ultrastructural analysis (SEM/TEM)	Reduction in cyst weight vs. untreated control (ABZ *P* < 0.001; thymol 20 days *P* < 0.01; thymol 10 days *P* < 0.05). No significant differences between thymol groups and ABZ (*P* > 0.05). Pronounced germinal layer damage with 12 h dosing interval	Comparable to ABZ (25 mg/kg/day)	Maggiore et al. [66]
*E. multilocularis*	CF-1 Mice	Thymol; Thymol + ABZ	40 mg/kg 40 + 5 mg/kg	20 days	Metacestode weight measurement; protoscoleces viability: methylene blue; ultrastructural analysis: SEM/TEM; Plasma enzyme activity (AP, GOT, GPT).	Metacestode weight reduction relative to untreated control: ABZ: 48% reduction (*P* < 0.05 vs. control); thymol: 60% reduction (*P* < 0.05 vs. control); thymol + ABZ: 83% reduction (*P* < 0.001 vs. control). Decreased protoscolex viability; severe germinal layer and microtriche damage were observed, especially with combination group; No significant changes in plasma enzyme levels were detected.	Thymol comparable to ABZ; combination more effective than either monotherapy	Albani et al. [67]
*E. granulosus*	CF-1 Mice	Carvacrol	40 mg/kg	20 days	Cyst weight measurement; ultrastructural analysis (SEM/TEM).	Cyst weight reduction: Carvacrol 72% reduction (*P* < 0.05 vs. untreated control group); not significantly different from ABZ (*P* > 0.05). Structural alterations of the germinal layer and parasite ultrastructure were observed.	Comparable to ABZ (73% reduction at 25 mg/kg)	Fabbri et al. [60]
*E. multilocularis*	Mice	Carvacrol; Carvacrol + ABZ	40 mg/kg; 40 + 25 mg/kg	30 days	Metacestode weight measurement; ultrastructural analysis (SEM); plasma enzyme activity (ALP, GGT, GOT, GPT)	Metacestode weight reduction: carvacrol: 28% reduction (*P* > 0.05 vs. control); ABZ: 50% reduction (*P* > 0.05 vs. control); carvacrol + ABZ: 83% reduction (*P* < 0.01 vs. control). Extensive germinal layer collapse with combination. significant alterations in plasma enzyme levels were reported.	Combination strongly more effective than either monotherapy	Lopez et al. [63]
*E. granulosus*	Mice	Thymol; Carvacrol; Thymol + carvacrol	40 mg/kg each	30 days	Cyst weight (median, IQR); ultrastructural analysis (SEM).	Significant reduction in cyst weight with ABZ, thymol and carvacrol monotherapies (*P* < 0.05 vs. untreated control); combination at equal doses showed a non-significant trend toward reduction vs. monotherapies (*P* > 0.05). Ultrastructural analysis revealed germinal layer alterations across treatment groups.	Thymol and carvacrol alone more effective than ABZ (25 mg/kg); combination less effective than ABZ	Albani et al. [65]

IQR: interquartile range

Carvacrol likewise exhibited in vivo anti-echinococcal activity. In *E. granulosus* infected mice, oral administration of carvacrol considerably reduced cyst weight, achieving an efficacy comparable to that of ABZ and causing similar tegumental damage [60]. In *E. multilocularis* models, carvacrol monotherapy achieved moderate reductions in parasite burden. However, when combined with ABZ, therapeutic efficacy markedly improved, with substantial decreases in metacestode biomass and severe structural collapse, without evidence of hepatic toxicity [63].

Comparative investigations indicate that thymol and carvacrol exhibit similar efficacy when administered as single agents. However, their co-administration does not consistently enhance anti-echinococcal activity. Equal-dose combinations did not outperform individual compounds, suggesting that pharmacokinetic interactions and dose ratios may critically influence therapeutic outcomes [65].

Overall, in vivo evidence supports thymol and carvacrol as complementary anti-echinococcal agents. Their ability to induce degenerative architectural changes in parasite tissues is consistent with in vitro observations. Nonetheless, heterogeneity in experimental design, treatment duration, dosing regimens, and infection models limit direct comparability across studies. The implementation of standardized experimental protocols will be essential to more accurately define their therapeutic potential and optimize rational combination strategies.

### Methodological Heterogeneity and Implications for Evidence Interpretation

Interpretation of anti-echinococcal efficacy requires caution because methodological heterogeneity across studies may substantially influence apparent potency estimates and translational relevance. Included studies use diverse viability endpoints, including dye exclusion assays (eosin or methylene blue), motility-based assessments, morphological scoring, ultrastructural observations, enzyme leakage assays, and TUNEL-based readouts. Because these endpoints capture distinct biological phenomena membrane integrity, metabolic activity, structural damage, or cell death they are not directly interchangeable and may yield different efficacy estimates. Similar concerns regarding endpoint heterogeneity affecting antiparasitic drug comparisons have been highlighted in helminth and antimicrobial screening literature [6, 61, 68].

Exposure conditions also vary markedly among studies, with treatment durations ranging from minutes to weeks and concentrations spanning wide micromolar and mass-based ranges [56, 57, 61]. Such variation complicates interpretation of relative potency, as rapid scolicidal activity observed under short-term high-dose exposure may not be directly comparable with effects measured under prolonged lower-dose exposure [56, 57, 65].

An additional source of heterogeneity concerns grouping distinct parasite stages within efficacy interpretations. Protoscoleces, germinal layer cells and metacestodes differ substantially in structure, permeability barriers, metabolic activity and drug susceptibility [68, 65]. Consequently, activity observed in one developmental stage should not be assumed to extrapolate directly to others. Similar stage-dependent susceptibility has been recognized as a major determinant of interpretation in experimental cestode chemotherapy models [68, 69].

Collectively, this heterogeneity limits direct cross-study comparisons and precludes robust conclusions regarding comparative potency or translational superiority. Greater harmonization of stage-specific models, viability endpoints and reporting standards will be essential for improving reproducibility and supporting future quantitative synthesis.

### Mechanisms Underlying the Anti-echinococcal Activity of Thymol and Carvacrol

**Fig. 3:**
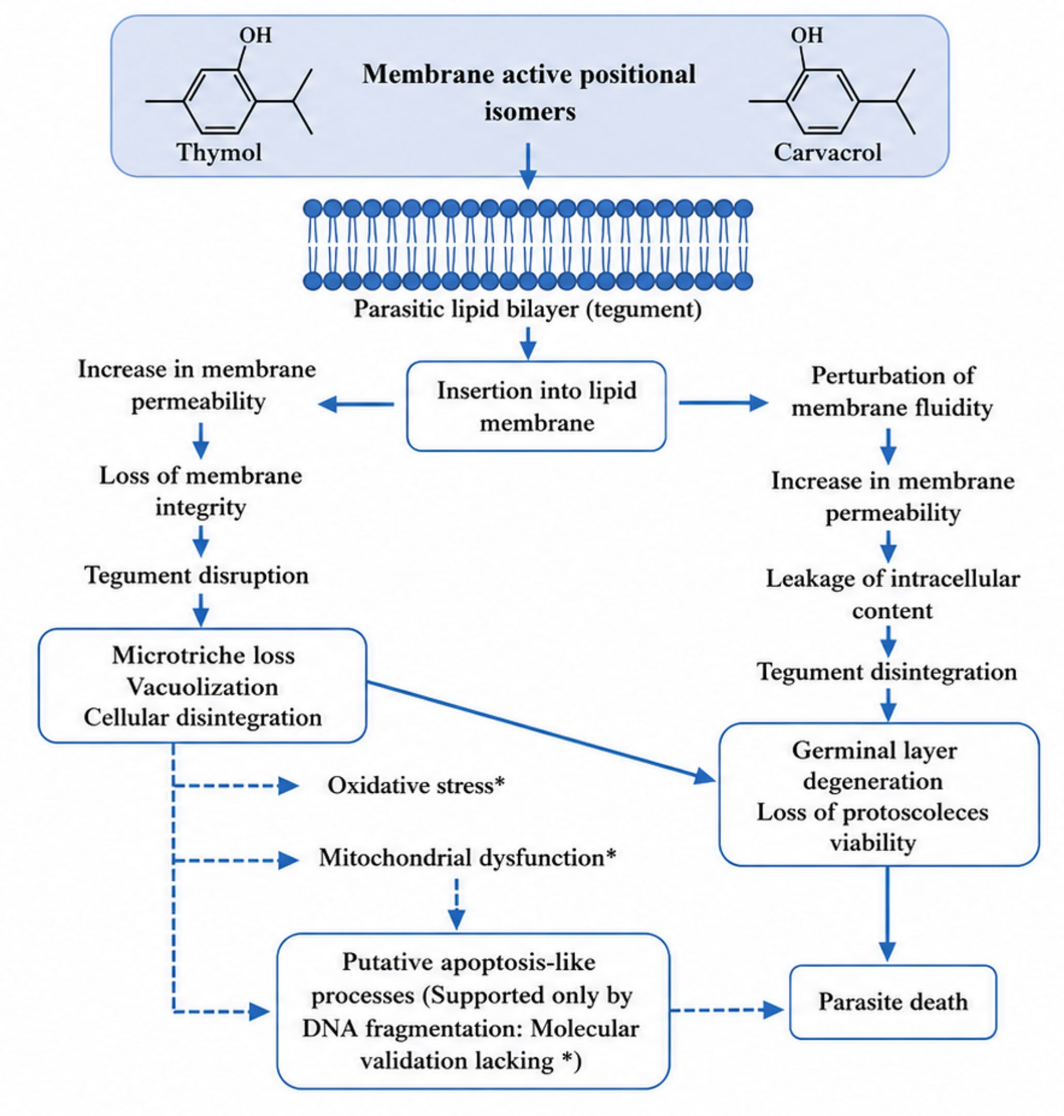
Schematic representation of the proposed anti-echinococcal mechanisms of thymol and carvacrol. Solid arrows represent effects supported by available experimental evidence, whereas dashed arrows with * indicate hypothetical pathways extrapolated from studies on plant-derived monoterpenes and essential oils.

Experimental evidence indicates that thymol and carvacrol exert anti-echinococcal activity through complementary structural and functional disturbances affecting larval stages (protoscoleces and metacestodes) of *E. granulosus* and *E. multilocularis*. The earliest and most consistently documented events occur at the tegumental membrane, where these hydrophobic monoterpenes incorporate into lipid bilayers, destabilize membrane organization, and initiate progressive degenerative changes. Subsequent damage extends to the germinal layer and internal tissues, ultimately leading to loss of parasite viability. While apoptosis-like pathways remain only partially supported by indirect evidence (mainly DNA fragmentation) and are not yet mechanistically validated in Echinococcus exposed to thymol or carvacrol. Importantly, no direct evidence currently demonstrates caspase-like activity, reactive oxygen species generation, mitochondrial membrane depolarization, or ATP depletion in Echinococcus exposed to thymol or carvacrol. A conceptual framework integrating experimentally supported and putative mechanisms is presented in Fig. 3.

### Membrane and Tegument Disruption

The primary and most consistently documented mode of action of thymol and carvacrol in *Echinococcus* spp. involves disruption of cell membranes. Due to their hydrophobic nature, these monoterpenoid phenols are thought to incorporate into lipid bilayers, which may alter membrane organization and function [59, 57]. This interaction may destabilize phospholipids and membrane-associated proteins, potentially resulting in increased permeability and partial loss of selective barrier integrity. Scanning and transmission electron microscopy (SEM and TEM) have demonstrated significant tegumental alterations in *E. granulosus* and *E. multilocularis* after exposure to these compounds. Observed alterations include deformation and detachment of microtriches, membrane blebbing, extensive vacuolization, surface irregularities, and, in advanced stages, complete tegument rupture [40, 66, 65]. Such damage is associated with leakage of intracellular contents, osmotic imbalance, and rapid loss of protoscoleces viability. The strong affinity of both compounds for lipid membranes is consistent with their physicochemical properties, notably high octanol/water partition coefficients, which favor deep incorporation into hydrophobic membrane regions [70]. Tegumental destabilization may not confined to the parasite surface and could precede broader intracellular degeneration, suggesting that membrane disruption could represent an early event preceding broader structural degeneration.

### Ultrastructural and Morphological Alterations

Beyond surface disruption, thymol and carvacrol have been associated with extensive ultrastructural alterations within parasite internal tissues. Electron microscopy analyses have revealed pronounced cytoplasmic vacuolization, mitochondrial swelling, parenchymal disorganization, and fragmentation of internal structures in treated protoscoleces [40, 65]. In metacestodes, the germinal layer undergoes marked degeneration, characterized by collapsed cellular architecture, areas of tissue disintegration, and loss of structural integrity [66]. These observations suggest that these monoterpenes may impair essential biological functions, such as nutrient absorption, tissue renewal, and developmental progression. They may also interfere with cyst maturation and protoscoleces formation.

### Metabolic Interference

Metabolic consequences of thymol and carvacrol exposure in *Echinococcus s*pp. are not well characterized. Nevertheless, structural damage induced by these compounds is suggestive of potential metabolic alterations. However, direct evidence for ATP depletion or mitochondrial bioenergetic collapse is currently lacking. Experimental evidence suggests that thymol exposure may be associated with increased release of gamma-glutamyl transferase (GGT), a membrane-associated enzyme involved in glutathione metabolism, into the culture medium of treated protoscoleces and metacestodes [57, 62]. The time-dependent increase in extracellular GGT activity may reflect membrane destabilization and enzymatic leakage, which provides functional support for metabolic interference beyond purely morphological disruption. Whether similar enzymatic alterations occur following carvacrol exposure remains to be determined. Direct measurements of ATP depleion, mitochondrial bioenergetic collapse, or targeted inhibition of specific metabolic pathways in response to either compound have not yet been systematically evaluated and require dedicated experimental validation.

### Preliminary Evidence Suggestive of Apoptosis-Like Responses

Current evidence is suggestive of apoptosis-like responses rather than sufficient to establish canonical apoptotic signaling. Although such responses may contribute to parasite death, evidence for their involvement in thymol- or carvacrol-treated *Echinococcus* remains incomplete and largely indirect. Support is currently limited primarily to TUNEL-detected DNA fragmentation, whereas molecular hallmarks required to validate apoptotic signaling pathways have not been demonstrated. Current evidence is suggestive of apoptosis-like responses rather than sufficient to establish canonical apoptotic signaling. Although such responses may contribute to parasite death, evidence for their involvement in thymol- or carvacrol-treated *Echinococcus* remains incomplete and largely indirect. Support is currently limited primarily to TUNEL-detected DNA fragmentation, but TUNEL positivity alone does not establish apoptosis, as it may also reflect necrosis-associated or nonspecific DNA damage. Molecular hallmarks required to validate apoptotic signaling pathways have not been demonstrated. Induction of apoptosis, or programmed cell death, represents an important cellular response to severe structural and biochemical stress. It is a well-recognized mechanism of action for several antiparasitic and antimicrobial agents [71]. In this context, apoptosis-like processes are considered potential mechanisms contributing to *Echinococcus* elimination. Caspases, particularly caspase-3, are established central mediators of apoptotic signaling pathways in many organisms [72].

In *Echinococcus* spp., apoptosis-like events following exposure to thymol and carvacrol have been partially documented. TUNEL analysis of protoscoleces treated with thymol revealed DNA fragmentation in situ as early as 8 h post-incubation [57]. Similarly, exposure to carvacrol resulted in clear TUNEL positive nuclei compared to untreated controls, indicating nuclear DNA fragmentation consistent with apoptosis-like cell death [60]. In both studies, TUNEL positivity was more pronounced in parasites with tegumental disruption, suggesting that severe structural damage may precede or accompany the activation of cell death pathways. These findings provide direct evidence of DNA fragmentation in protoscoleces treated with thymol or carvacrol. However, additional components of apoptotic signaling pathway such as caspase-3-like activation, mitochondrial membrane potential collapse, cytochrome c release, or reactive oxygen species (ROS) quantification, have not yet been evaluated in thymol or carvacrol treated *Echinococcus* models. Thus, while experimental data support the occurrence of apoptosis-like nuclear alterations, the precise molecular cascade remains incompletely characterized.

This limitation is particularly relevant, given that multiple studies using plant-derived extracts provide evidence consistent with apoptosis-like responses in *Echinococcus*. Studies using ethanolic extracts of *Taraxacum officinale* and chloroform extracts of *Astragalus ecbatanus* indicate dose-dependent activation of caspase-3-like enzymes, accompanied by morphological hallmarks consistent with programmed cell death, including chromatin condensation, membrane blebbing, and cytoplasmic vacuolization [73, 74]. Similar apoptotic responses have been described for *Ferula macrecolea* essential oil, which triggered up to 48.3% caspase-3-like activation in treated protoscoleces [75], and for *Astragalus brachycalyx* which stimulated caspase-3-like activity by 8.8% to 29.6% [76]. More recently, *Astragalus onobrychis* extract was found to significantly activate caspase-3-like enzymes and upregulate genes associated with DNA damage response, such as EgATM and EgP53. This provides molecular evidence of an intrinsic apoptotic response in *E. granulosus* [77].

Evidence from other protozoan parasites suggests that thymol and carvacrol may affect oxidative stress and mitochondrial-associated death pathways, however, these mechanisms have not been experimentally demonstrated in *Echinococcus* and should therefore not be interpreted as established mechanisms in this system. In these organisms, mechanisms typically involve oxidative stress characterized by reactive oxygen species generation, followed by loss of mitochondrial membrane potential, the release of cytochrome c, and activation of caspase-dependent pathways [18, 78, 79]. The consistency of these findings across phylogenetically distinct parasites suggests that thymol and carvacrol may possess intrinsic pro-apoptotic activity across diverse protozoan. Their membrane partitioning properties and effects on mitochondrial integrity observed in other parasite systems suggest a possible contribution to putative apoptosis-like mechanisms.

These observations provide direct evidence of apoptosis-like DNA fragmentation in thymol and carvacrol treated protoscoleces, while involvement of complete apoptotic signaling pathways remains inferential. Importantly, no direct evidence currently demonstrates caspase-like activity, reactive oxygen species generation, mitochondrial membrane depolarization, or ATP depletion in *Echinococcus* exposed to thymol or carvacrol. Comprehensive molecular validation, including assessment of these pathways, will be necessary to determine whether canonical or alternative programmed cell death pathways are implicated. At present, these pathways should be regarded as mechanistic hypotheses rather than experimentally validated components of anti-echinococcal activity.

To systematically bridge the gap between morphological observations and molecular validation, key mechanistic evidence gaps and corresponding experimental strategies are outlined in Table 3.

**Table 3:** Mechanistic evidence gaps preventing validation of apoptosis-like responses

Evidence gap	Mechanism	Experimental approach	Specific endpoint
No direct measurement of oxidative stress	Oxidative stress induction	DCFH-DA fluorescence assay; lipid peroxidation (MDA levels); SOD activity measurement	Quantify intracellular ROS production and oxidative damage
Lack of mitochondrial functional data	Mitochondrial dysfunction	JC-1 or TMRE assay	Assess mitochondrial membrane potential (ΔΨm) depolarization
Absence of caspase confirmation	Caspase-dependent apoptosis	Fluorometric caspase-3-like activity assay	Confirm activation of apoptotic execution pathways
DNA fragmentation not linked to viability	Apoptosis-like nuclear damage	Quantitative TUNEL assay (flow cytometry-based)	Determine extent and kinetics of DNA fragmentation
Limited molecular pathway characterization	Stress-response and intrinsic apoptosis signaling	qPCR analysis of EgATM, EgP53, and stress-related genes	Evaluate activation of intrinsic apoptotic pathways
Absence of direct ATP and mitochondrial bioenergetic profiling	Energy metabolism disruption	ATP quantification assay; mitochondrial assay; GGT release measurement	Assess ATP depletion, mitochondrial dysfunction, and enzymatic leakage
Lack of standardized viability readouts	Standardized parasite viability	Resazurin-based or ATP-based viability assays	Improve reproducibility and inter study comparability

### Mechanism of Albendazole

Albendazole, a benzimidazole carbamate, remains the reference drug for the treatment of CE and AE. Following oral administration, ABZ is rapidly metabolized in the liver to its active metabolite, ABZ sulfoxide, which is responsible for the systemic antiparasitic activity. Its primary mode of action involves selective binding to parasite β-tubulin, which inhibits microtubule polymerization and subsequently disrupts cytoskeletal organization as well as microtubule-dependent intracellular transport [80, 81]. Consequently, essential cellular processes such as glucose uptake, vesicular trafficking, and nutrient absorption are progressively impaired, resulting in glycogen reserves depletion and reduced ATP production [82, 83]. In *Echinococcus* spp., these metabolic disturbances progressively compromise parasite viability. However, overall effect is predominantly parasitostatic rather than parasiticidal particularly in *E. multilocularis* infections, where continuous and often lifelong drug exposure is required to prevent parasite regrowth [8]. Given ABZ’s parasitostatic profile and the necessity of prolonged exposure, examining the mechanistic differences between ABZ and membrane-active monoterpenoid phenols could provide insights into their potential complementary or synergistic roles, which is explored in the following comparative analysis.

### Comparative Analysis

Understanding the established mechanism of action of ABZ and comparing it with those proposed for thymol and carvacrol is useful to assess the therapeutic relevance of these monoterpenoid phenols as potential adjunctive agents in the treatment of echinococcosis. While ABZ remains the cornerstone of current chemotherapy, its pharmacokinetic limitations and predominantly parasitostatic profile contrast with the rapid membrane-directed effects observed for thymol and carvacrol in experimental models. An additional mechanistic hypothesis is that membrane-active monoterpenes may enhance ABZ access at the parasite interface by altering permeability barriers, including the laminated cyst wall or parasite membranes. Such effects could, in principle, contribute to pharmacokinetic or pharmacodynamic complementarity beyond direct antiparasitic synergy. However, this remains speculative, as no studies have directly evaluated whether thymol or carvacrol improve intracystic ABZ penetration or local drug exposure [19, 65, 68]. These mechanistic differences help to clarify variations in efficacy patterns observed across in vitro and in vivo studies and provide a conceptual basis to interpret potential synergistic interactions when these compounds are used in combination. Important pharmacological distinctions emerge, particularly regarding activation requirements, kinetics of action, functional outcomes, and resistance dynamics. These major pharmacological distinctions between ABZ and membrane-active monoterpenoid phenols are summarized in Table 4.

**Table 4:** Comparative pharmacological and mechanistic characteristics of albendazole and thymol/carvacrol in echinococcosis

Parameter	Albendazole (ABZ)	Thymol /Carvacrol	Implications
Active form	Albendazole sulfoxide (systemic active metabolite)	Free phenolic form (no metabolic activation required)	Monoterpenes act immediately, whereas ABZ requires hepatic biotransformation
Level of evidence	Extensive clinical and preclinical evidence	Mainly in *vitro* and in vivo experimental evidence	Different validation levels must be considered when interpreting efficacy
Primary target and mechanism	Selective binding to β-tubulin→ inhibition of microtubule polymerization	Interaction with lipid membranes leading to increased permeability and membrane destabilization	Divergent mechanisms may support therapeutic complementarity
Selectivity of action	High affinity for parasite β-tubulin	Broad membrane interaction; host selectivity not fully characterized	Viability in selectivity may influence therapeutic index and resistance dynamics
Kinetics of action	Slow onset; requires prolonged exposure	Rapid and dose-dependent activity	Rapid scolicidal action may complement slow-acting drugs
Functional outcome	Metabolic disruption and energy depletion (mainly parasitostatic)	Rapid structural damage, scolicidal and cysticidal effects (experimental models)	Combining parasitostatic and parasiticidal effects may enhance parasite control
Pharmaco kinetic limitations	Variable cyst penetration; prolonged treatment required	Limited solubility and bioavailability; intracystic penetration poorly characterized; improved formulations under investigation	Optimized delivery systems are required to enhance intracystic exposure
Resistance potential	Documented in other helminths via β-tubulin mutations	Not yet characterized in *Echinococcus* spp.	Distinct selective pressures may affect long-term sustainability

Data on thymol and carvacrol are primarily derived from experimental studies. Direct clinical comparisons with albendazole in human echinococcosis are currently lacking

Beyond these mechanistic differences, membrane-active monoterpenoid phenols may exert a selective pressure that differs from that of ABZ, whose single-target mechanism has been associated with the development of resistance in other helminths. Whether such mechanistic divergence influences the emergence of resistance in *Echinococcus* spp. remains unresolved and warrants systematic investigation.

### Translational Pharmacokinetic Limitations and Intracystic Exposure Challenges

A critical issue in the translation of thymol and carvacrol into clinical practice concerns whether the concentrations shown to be effective in vitro can be realistically achieved in vivo, particularly within hydatid cysts. While membrane disruption and apoptosis-like effects have been consistently observed in vitro, the in vivo relevance of these mechanisms depends on whether sufficient intracystic concentrations are attained. Most in vitro studies report protoscolicidal activity at concentrations between 10 and 250 µg/mL. However, pharmacokinetic investigations indicate that, following oral administration, monoterpenoid phenols undergo extensive first-pass metabolism and rapid phase II conjugation, limiting systemic persistence of parent compound [84, 85].

Available pharmacokinetic data indicate that peak systemic concentrations of thymol in animal models generally remain within the nanogram to low microgram-per-milliliter range, typically below 4 µg/mL, depending on species and formulation [86]. These levels are therefore lower than those commonly used in protoscolicidal in vitro assays. Although thymol is distributed across multiple tissues in animal species, it is rapidly conjugated to sulfate and glucuronide metabolites, resulting in limited persistence of the free parent compound in systemic circulation [86]. In humans, no free thymol has been detected in plasma following oral administration, while only thymol sulfate was measurable at nanogram-per-milliliter concentrations [84]. These findings align with interspecies data on first-pass metabolism, emphasizing the challenge of achieving sufficient unconjugated exposure to replicate in vitro effects [85].

Interpretation of anti-echinococcal efficacy also requires consideration of differences between in vitro exposure concentrations and in vivo dosing regimens. In vitro activity is often observed at micromolar or µg/mL concentrations under direct parasite exposure, whereas in vivo studies generally employ systemic mg/kg dosing influenced by absorption, distribution, metabolism and tissue penetration. Because these exposure metrics are not directly equivalent, apparent potency observed in vitro may not readily predict in vivo efficacy. This distinction is particularly relevant when considering translational interpretation of scolicidal concentrations relative to achievable tissue or intracystic exposure.

Although carvacrol fulfils classical drug-likeness criteria (Lipinski, Veber, and Egan parameters), experimental data indicate incomplete gastrointestinal absorption and extensive hepatic metabolism [87]. Animal studies further suggest that a substantial proportion of orally administered carvacrol remains in the gastrointestinal tract, with significant urinary excretion of phase I and phase II metabolites, including glucuronide and sulfate conjugates [51, 88]. Such rapid biotransformation and elimination may limit sustained free concentrations in plasma and peripheral tissues. Taken together, these data raise important questions about whether concentrations effective in scolicidal in vitro assays can realistically be achieved and maintained at relevant target sites in vivo.

Another important consideration from a clinical perspective is the ability of candidate compounds to penetrate the laminated layer of hydatid cysts and achieve effective intracystic exposure. In CE, target-site drug concentrations within the cyst are critical determinants of efficacy. Systematic pharmacokinetic analyses of patient data revealed substantial inter-individual variability in intracystic ABZ sulfoxide concentrations and, importantly, no correlation between plasma and intracystic levels [89]. These findings suggest that systemic exposure alone is not a reliable predictor of intracystic penetration. Given that even ABZ, the current first-line therapy, shows unpredictable distribution, the absence of direct intracystic pharmacokinetic data for thymol and carvacrol represents a significant translational limitation. Without such data, definitive conclusions regarding their therapeutic feasibility in established cystic lesions remain premature.

In the context of combination therapy, potential pharmacokinetic interactions should also be considered. ABZ undergoes hepatic biotransformation to ABZ sulfoxide through flavin monooxygenase and cytochrome P450-dependent pathways, including CYP3A isoforms. This has been observed in mammalian liver microsomal systems [90]. Studies using human liver microsomes have shown that thymol and carvacrol are predominantly metabolized by CYP2A6, with carvacrol undergoing extensive oxidative metabolism via CYP-mediated pathways [88]. As these compounds depend on CYP-mediated clearance, theoretical interactions affecting first-pass metabolism, systemic exposure, or biliary excretion cannot be excluded. Such interactions may alter pharmacokinetic profiles and potentially complicate interpretation of apparent “synergistic” effects observed in experimental models. In the absence of dedicated pharmacokinetic interaction studies and intracystic exposure measurements, conclusions regarding therapeutic synergy remain provisional.

Overall, although in vitro mechanistic studies suggest that thymol and carvacrol have promising protoscolicidal and apoptosis-inducing properties, there is limited translational pharmacokinetic data. Key uncertainties that must be addressed in future studies to validate the in vivo relevance of the observed mechanisms include achievable systemic and intracystic concentrations, metabolic stability, and tissue penetration.

### Perspectives and Future Directions

Despite significant advances in the understanding of the biological effects of thymol and carvacrol against *Echinococcus* spp., there are still several critical gaps that must be addressed before their potential therapeutic relevance in the treatment of echinococcosis can be adequately assessed. Current evidence is largely derived from in vitro and limited in vivo models, so direct clinical translation remains premature. Future research should prioritize the generation of robust preclinical data integrating pharmacokinetic, pharmacodynamic, and toxicological evaluations to better support translational development.

One major challenge concerns the poor aqueous solubility and limited bioavailability of monoterpenoid phenols, which may restrict effective concentrations from being achieved at the site of infection. Developing optimized formulations, including nanoencapsulation, controlled- release delivery systems, or lipid-based carriers, could enhance systemic exposure and intracystic penetration while mitigating dose-dependent toxicity. Such approaches would also facilitate a more reliable evaluation of efficacy in relevant animal models of CE and AE.

Systematic investigation of combination therapies with ABZ represents another important research direction. Given their distinct and potentially complementary mechanisms of action, the co-administration of ABZ with monoterpenoid phenols warrants further exploration. Future studies should aim to define optimal dosing regimens, treatment durations, and sequences of administration, while carefully assessing additive or synergistic effects, as well as safety profiles. Moreover, combining a microtubule-targeting agent with membrane-active monoterpenoid phenols could conceptually alter the selective pressures imposed on the parasite, potentially impacting resistance development. However, this hypothesis remains speculative and requires dedicated experimental validation.

Another limitation of the currently available evidence concerns the methodological quality of primary studies. Many studies involve small experimental sample sizes, heterogeneous viability assays, inconsistent exposure protocols, and limited blinding or standardized outcome reporting. These factors complicate comparability and may introduce bias into efficacy estimates. Future studies would benefit from standardized reporting and risk-of-bias conscious experimental design. In addition, because study screening and data extraction were performed by a single reviewer, some risk of selection bias cannot be excluded.

Finally, advanced mechanistic studies employing imaging techniques, ultrastructural analyses, and molecular approaches are necessary to refine the understanding of how parasites respond to monoterpenoid phenols. Standardization of experimental protocols and outcome measures across studies would greatly improve comparability and reproducibility. Collectively, these efforts will be crucial to determine whether thymol and carvacrol can progress from experimental antiparasitic agents to scientifically justified adjuncts in future therapeutic strategies for echinococcosis, particularly in settings where therapeutic options remain suboptimal. Translational frameworks integrating optimized formulations, combination therapies, and mechanistic validation will be essential for bridging the gap between laboratory findings and real-world clinical application.

## Conclusion

This narrative review consolidates experimental evidence supporting the anthelmintic activity of thymol and carvacrol against *E. granulosus and E. multilocularis*, with particular emphasis on their membrane-disruptive effects and rapid induction of structural damage in experimental models. Their scolicidal and cysticidal activities contrast with the microtubule targeting, predominantly parasitostatic effect of ABZ, the current cornerstone of echinococcosis chemotherapy. This divergence provides a biologically plausible rationale for potential complementarity and combination strategies. Nevertheless, significant translational gaps remain. Current data are largely restricted to in vitro and a limited number of in vivo investigations. Poor aqueous solubility, limited bioavailability, methodological heterogeneity and lack of study-quality assessment, and the absence of standardized endpoints prevent clear therapeutic positioning of these monoterpenoid phenols. Progress will require harmonized protoscoleces viability assays, pharmacokinetic profiling in relevant animal models, and systematic evaluation of combination regimens with ABZ. Advancing thymol and carvacrol beyond experimental compounds will depend on reproducible preclinical validation, formulation optimization, and rigorous safety assessment. With robust supporting evidence, natural adjunct strategies may ultimately contribute to expanding therapeutic options, particularly in endemic settings where long-term benzimidazole therapy remains suboptimal. The complete absence of clinical studies remains a major translational barrier.

## Data Availability

No datasets were generated or analysed during the current study.

## Abbreviations

ABZ: Albendazole
ABZSO: Albendazole sulfoxide
AE: Alveolar echinococcosis
ALP: Alkaline phosphatase
ALT (GPT): Alanine aminotransferase
AST (GOT): Aspartate aminotransferase
ATP: Adenosine triphosphate
BZM / BZMs: Benzimidazole (methyl carbamates)
CA: Carvacrol
CE: Cystic echinococcosis
CYP: Cytochrome P450
DALYs: Disability-adjusted life years
DCFH-DA: 2′,7′-Dichlorodihydrofluorescein diacetate
FDA: Food and Drug Administration
GGT: Gamma-glutamyl transferase
GRAS: Generally Recognized As Safe
IQR: Interquartile range
JC-1: 5,5′,6,6′-Tetrachloro-1,1′,3,3′-tetraethylbenzimidazolylcarbocyanine iodide
LD50: Median lethal dose
MAPK: Mitogen-activated protein kinase
MDA: Malondialdehyde
MBZ: Mebendazole
NF-κB: Nuclear factor kappa B
qPCR: Quantitative polymerase chain reaction
ROS: Reactive oxygen species
SEM: Scanning electron microscopy
SOD: Superoxide dismutase
TEM: Transmission electron microscopy
TMRE: Tetramethylrhodamine ethyl ester
TNF-α: Tumor necrosis factor alpha
TUNEL: Terminal deoxynucleotidyl transferase dUTP nick-end labeling
ΔΨm: Mitochondrial membrane potential
